# Mediating role of chiro-inositol metabolites on the effects of HLA-DR-expressing CD14 + monocytes in inflammatory bowel disease

**DOI:** 10.1186/s12876-024-03271-2

**Published:** 2024-06-17

**Authors:** Leichang Zhang, Pan Shen, Wei Ge, Wu Liao, Qinghua Luo, Chaofeng Li, Chuanyu Zhan, Xiao Yuan, Xiaonan Zhang, Xiaojun Yan

**Affiliations:** 1 Affiliated Hospital of Jiangxi College of TCM, Nanchang, Jiangxi 330000 China; 2 Jiangxi University of Traditional Chinese Medicine, Nanchang, Jiangxi 330000 China

**Keywords:** Inflammatory bowel disease, Immunity, Metabolite, Causal inference, Mendelian randomization analysis

## Abstract

**Background:**

Inflammatory bowel disease (IBD), a chronic inflammatory condition, is caused by several factors involving aberrant immune responses. Genetic factors are crucial in IBD occurrence. Mendelian randomization (MR) can offer a new perspective in understanding IBD’s genetic background.

**Methods:**

Single nucleotide polymorphisms (SNPs) were considered instrumental variables (IVs). We analyzed the relationship between 731 immunophenotypes, 1,400 metabolite phenotypes, and IBD. The total effect was decomposed into indirect and direct effects, and the ratio of the indirect effect to the total effect was calculated.

**Results:**

We identified the causal effects of HLA-DR-expressing CD14 + monocytes on IBD through MR analysis. The phenotype *“HLA-DR expression on CD14 + monocytes”* showed the strongest association among the selected 48 immune phenotypes. Chiro-inositol metabolites mediated the effect of CD14 + monocytes expressing HLA-DR on IBD. An increase in Chiro-inositol metabolites was associated with a reduced risk of IBD occurrence, accounting for 4.97%.

**Conclusion:**

Our findings revealed a new pathway by which HLA-DR-expressing CD14 + monocytes indirectly reduced the risk of IBD occurrence by increasing the levels of Chiro-inositol metabolites. The results provided a new perspective on the immunoregulatory mechanisms underlying IBD, laying a theoretical foundation for developing new therapeutic targets in the future.

**Supplementary Information:**

The online version contains supplementary material available at 10.1186/s12876-024-03271-2.

## Introduction

Inflammatory bowel disease (IBD), including Crohn’s disease (CD) and ulcerative colitis (UC), is a chronic inflammatory disorder of the colon and small intestine, which manifests with wide-ranging symptoms, such as abdominal pain, diarrhea, and weight loss [1–3]. IBD’s global incidence is rapidly increasing, exerting massive pressure on public health systems worldwide [4]. An increased risk of disease onset has been observed among first-degree relatives of patients with IBD, which highlights the genetic component of the disease and underscores the necessity to investigate its genetic patterns and environmental triggers [5, 6]. Existing mainstream treatment regimens are associated with systemic side effects [7]; therefore, exploring new therapeutic targets is crucial.

At the core of IBD’s pathogenesis is immune system dysregulation, manifesting through abnormal responses of T cells, B cells, mast cells, activated neutrophils, and macrophages [8], contributing to chronic inflammation in IBD [9]. Therefore, research into the genetic and molecular mechanisms underpinning immune cell functions and their interaction in the context of IBD is necessary [10–13]. Genome-wide association studies (GWASs) have unveiled over 200 genomic loci linked to IBD, serving as a foundation for genetic inquiries [14]. These discoveries have facilitated exploration into the association of IBD with its causative factors [15–17]. In recent years, numerous studies have focused on exploring the role of immune cells in IBD, emphasizing the importance of immune cells in initiating or mitigating intestinal inflammation [9, 18–20]. Despite these insights enhancing our knowledge of IBD’s pathophysiology and inflammatory regulation, the quest for definitive causal associations at the population level remains.

Mendelian randomization (MR), leveraging genetic variants from GWASs as instrumental variables (IVs), is pivotal in bridging the gap between genetic predispositions and IBD by establishing causal links between genetic factors and this disease [21]. This offers novel insights into its pathogenesis and potential interventions, circumventing confounding factors typical in observational studies [22].

We collected genetic variants from large GWASs and analyzed the causal relationship between 731 immunophenotypes, 1400 metabolite phenotypes, and IBD. We investigated the effects of immune cells on IBD and the mediating role of metabolites through MR analysis. Our findings provide new insights for identifying potential diagnostic biomarkers and therapeutic targets.

## Methods

### Study design

We conducted a two-sample MR analysis to determine the causal relationship of immune cells with IBD. Single nucleotide polymorphisms (SNPs) were considered IVs to define these relationships. MR analysis is based on the following three assumptions: (1) genetic variations used as IVs are associated with immune traits; (2) IVs are independent of confounding factors; (3) IVs affect IBD only through their effect on immune traits and not through other pathways [23].

### Data sources

Data on IBD utilized were sourced from the FinnGen GWAS (https://r10.finngen.fi/, including 9,083 cases of IBD and 403,098 controls). IBD cases were identified using the FinnGen project’s strict definition (K11_IBD_STRICT), requiring confirmation in the Finnish National Drug Reimbursement Registry (KELA), which ensured high diagnostic accuracy and clinical relevance [24]. This approach is consistent with international guidelines provided by the European Crohn’s and Colitis Organisation (ECCO). According to ECCO, the diagnosis of CD involves a comprehensive evaluation including endoscopic, histopathological, and imaging findings to detect granulomatous inflammation and transmural lesions. The diagnosis of UC primarily relies on continuous colonic involvement and superficial inflammation, supported by histopathological findings, such as crypt architectural changes and mucosal inflammatory infiltrates [25].

We analyzed 731 immunophenotypes derived from 3,757 European individuals, including absolute cell counts, median fluorescence intensities, morphological parameters, and relative cell counts, spanning a spectrum of immune cells at various stages of maturation. Supplementary Table 1 presents the detailed immunophenotypes. We used 22 million SNPs, adjusted for factors such as sex and age, ensuring a robust analysis of genetic influences on these immune traits without cohort overlap, using data imputed from the Sardinian genetic reference [26].

Metabolites analyzed were sourced from the Canadian Longitudinal Study on Aging (CLSA) cohort, comprising a comprehensive array of 1,091 metabolite phenotypes and 309 metabolite phenotype ratios from 8,299 participants [27]. Supplementary Table 2 presents the detailed metabolite phenotypes. This rich dataset provided a solid foundation for investigating the potential metabolic factors affecting IBD pathogenesis.

### IV selection

We referred to the latest research findings and rigorously selected IVs suitable for MR analysis to ensure the accuracy and reliability of our evaluation [28]. SNPs were required to meet a genome-wide significance level (*p* < 5 × 10^− 8^). A clumping algorithm was utilized to avoid linkage disequilibrium effects (r^2^ = 0.001 and kb = 10,000). SNPs were selected based on their specific effect on immune cells and metabolites without directly affecting IBD. This ensured a sufficient F-statistic value to confirm the strength of the genetic instruments (F-statistics > 10) [29, 30]. PhenoScanner V2 (http://www.phenoscanner.medschl.cam.ac.uk/phenoscanner, accessed on 31 May 2023) was utilized to eliminate confounding factors among the positive results [31]. Our steps ensured the construction of a reliable framework for examining the genetic connections between immune cells, metabolites, and IBD and precise insights into the disease.

### Statistical analysis

An MR analysis was performed using the TwoSampleMR software package (version 0.5.6) in the R software (version 4.3.2) [32]. In our study, the inverse variance weighted (IVW) method was the primary analytical tool to explore the causal relationships among 731 immunophenotypes, 1,400 metabolite phenotypes, and IBD. This approach, involving a weighted regression of SNP-specific Wald ratios, allows for precisely assessing the causal effect of these variables on IBD. This highlights the foundational role of IVW in our investigation into the complex relationships within our dataset [33]. MR Egger [34], weighted median [35], and weighted mode were used as supplementary analyses to test the robustness of the results. MR-Egger analysis assesses the directional pleiotropy, capturing an average effect of genetic variation’s pleiotropy through its intercept. The weighted median approach is remarkable for its accuracy and resilience to pleiotropy. It reliably provides consistent estimates but a significant proportion of SNPs might not be valid IVs [36].

### Primary analysis

**Fig. 1:**
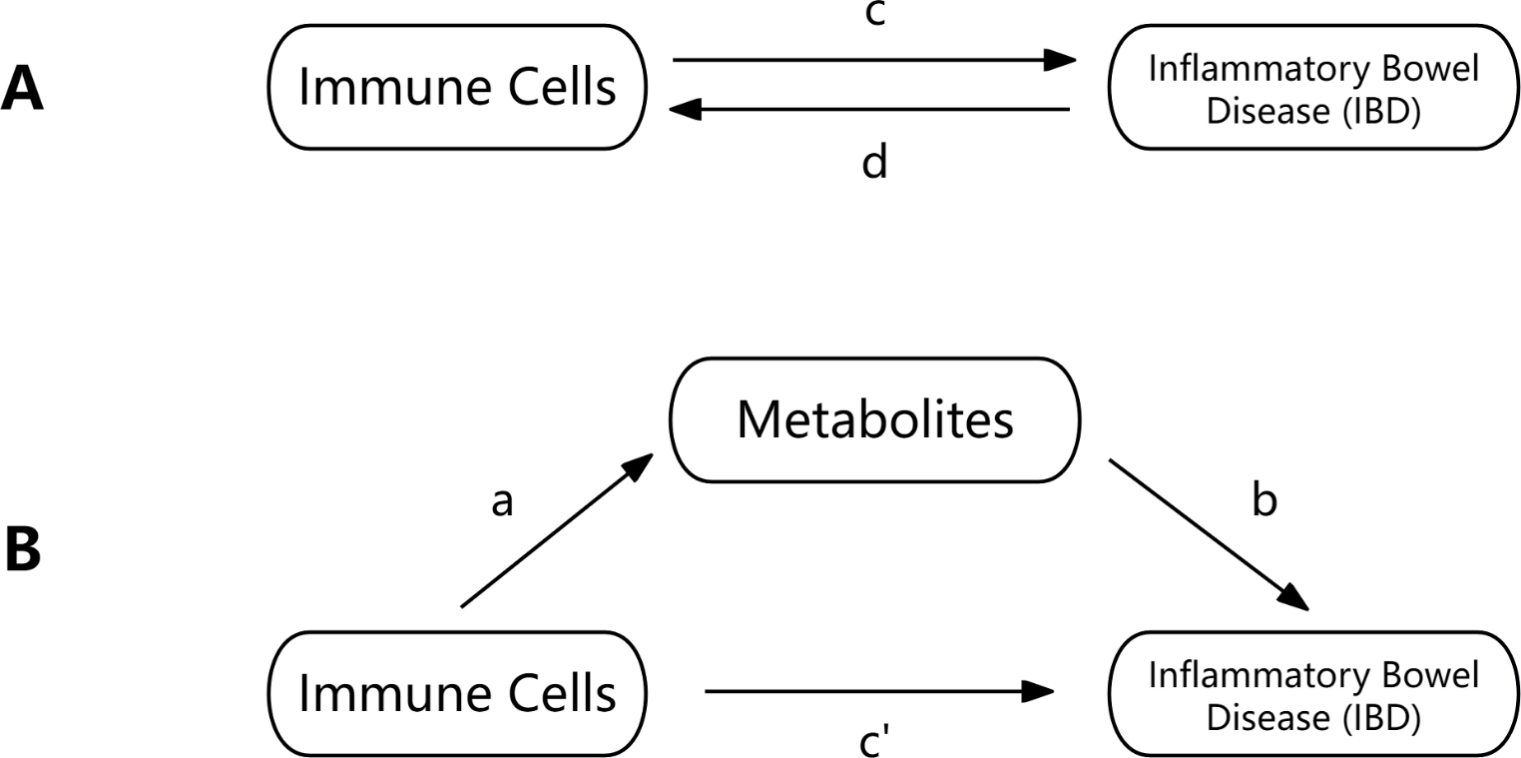
Relationships explored in this research. (**A**) The overall effect of immune cells on inflammatory bowel disease (IBD), with “c” representing the total effect using genetically predicted immune cells as the exposure and IBD as the outcome, and “d” indicating the converse. (**B**) Breaking down the total effect into: (i) an indirect effect through a two-step method (where “a” is the effect of immune cells on metabolites, and “b” is the effect of metabolites on IBD, calculated as “a × b”) and (ii) a direct effect (“c′ = c – a × b”), with the proportion mediated represented as the ratio of the indirect to the total effect

Figure 1 presents a diagrammatic overview of our analyses. We assessed the reciprocal causative relationships between immune cells and IBD through a two-sample bidirectional MR, termed the total effect in Fig. 1A.

We aimed to identify the immune cells with the most significant statistical association (smallest *p*-value) with IBD through cycling analysis of 731 immunophenotypes, focusing on their mediating effects. This approach allows targeted examination of the potential intermediaries between specific immune cell types and the pathogenesis of IBD.

### Mediation analysis

We expanded our analysis with mediation to assess if metabolites mediate immune cell function in IBD, as shown in Fig. 1B [37]. This analysis differentiated between the direct effect of immune cells on IBD and that channeled through metabolites. We quantified the mediated percentage by dividing the mediated effect by the total effect, applying the delta method for the corresponding 95% confidence intervals [38].

### Sensitivity analysis

The presence of reverse causation affects the outcomes of mediation effects. We employed the MR-Steiger test to verify the directionality from exposure to outcomes [39]. The Cochran’s Q statistic and funnel plots were used to evaluate heterogeneity among SNPs [40, 41]. A leave-one-out analysis was conducted to ensure the robustness of the results and any single SNP’s significant effect on the findings of MR analysis [42]. We explored the reciprocal effect of IBD on immune cell composition, utilizing the MR-PRESSO method for detecting outliers to mitigate bias from horizontal pleiotropy. This enhanced the credibility of our causal interpretations within the intricate relationship between the immune system and IBD [43].

## Results

### Association of HLA-DR-expressing CD14 + monocytes with IBD

Based on the IVW method, we investigated the causal effect of 731 immunophenotypes on IBD by a two-sample MR method. SNPs with a *p-*value < 5 × 10^− 8^ were selected as IVs, while palindromic and ambiguous SNPs were excluded to ensure clarity and accuracy.

Our analysis using the IVW method identified 48 immunophenotypes exhibiting significant associations (*p*-value < 0.05) with IBD from 731 immunophenotypes (Supplementary Table 3). We selected the immunophenotype most likely to exhibit significant associations for subsequent mediation analysis, specifically “*HLA-DR on CD14 + monocyte*,” demonstrating the smallest *p*-value (*p* = 3.86 × 10^− 6^), indicating a strong potential association. The odds ratio was 0.911 (95% CI = 0.876–0.948). No confounding factors were identified after uploading IVs onto PhenoScanner V2.

**Fig. 2:**
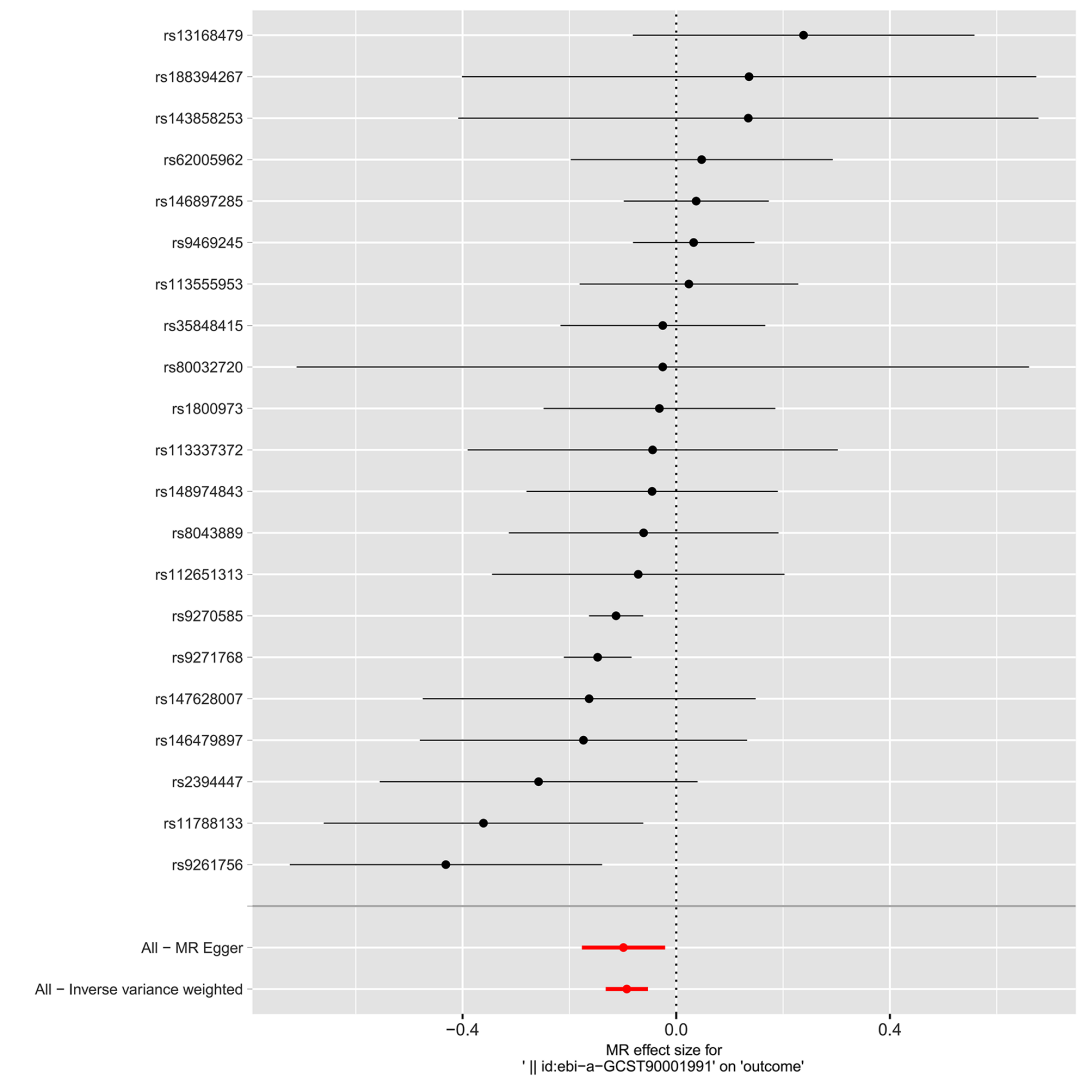
Forest plot shows the causal relationships of each single SNP with the total IBD risk

**Fig. 3:**
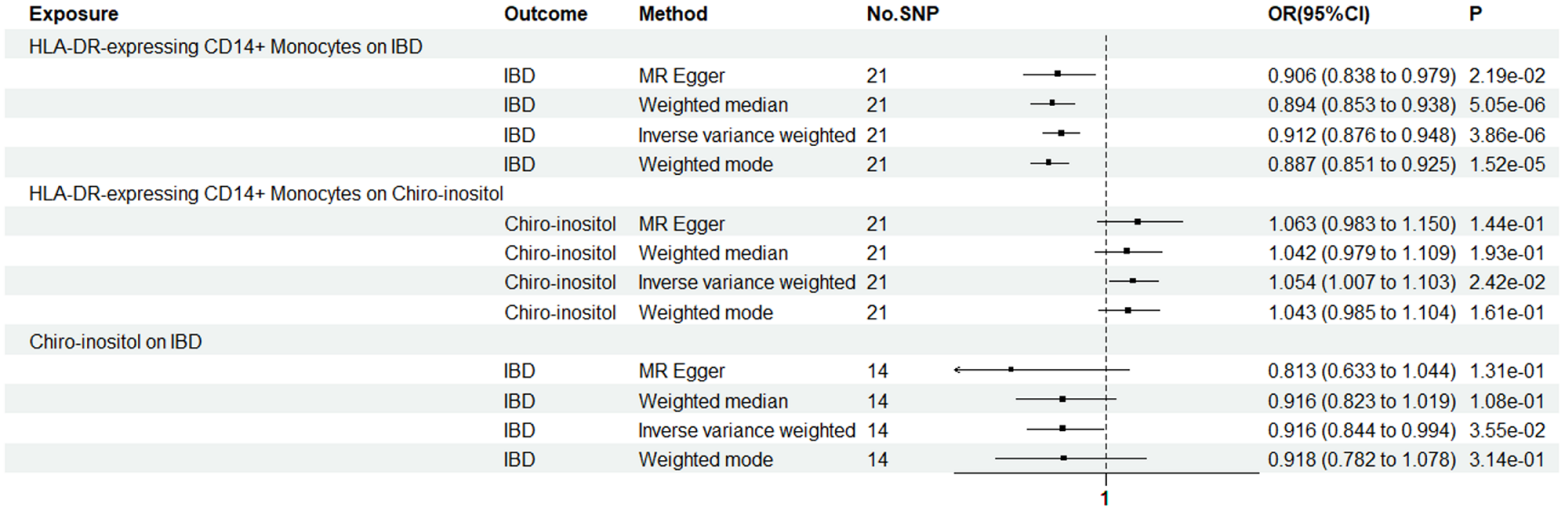
Forest plot shows the causal relationships among HLA-DR-expressing CD14 + monocytes, Chiro-inositol metabolites, and IBD

**Fig. 4:**
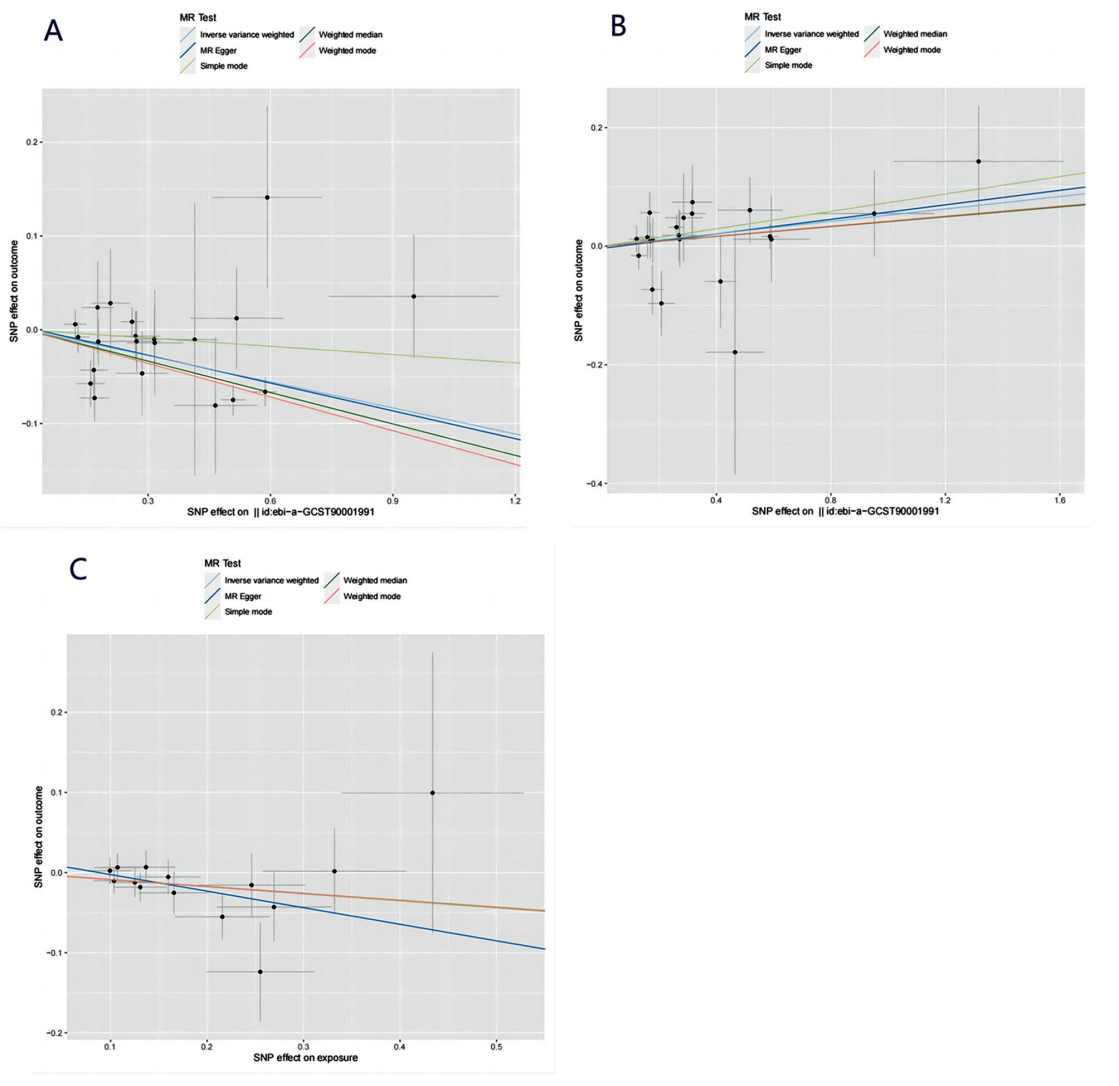
Scatter plot of the relationship among HLA-DR-expressing CD14 + monocytes, Chiro-inositol metabolites, and IBD. (**A**) HLA-DR-expressing CD14 + monocytes and IBD; (**B**) HLA-DR-expressing CD14 + monocytes and Chiro-inositol metabolites; (**C**) Chiro-inositol metabolites and IBD

IVW, MR-Egger, weighted median, and weighted mode were used to evaluate the causal relationships between HLA-DR-expressing CD14 + monocytes and IBD, as shown in Figs. 2 and 3, and 4. Evidence strongly supported a positive correlation between HLA-DR-expressing CD14 + monocytes and the incidence of IBD consistently across methodologies.

We conducted an MR-Steiger test (Supplementary Table 4) and a reverse MR analysis (Supplementary Table 5), with *P*_Steiger_ = 3.72 × 10^− 37^ and *P*_IVW for reverse MR analysis_ = 0.78 to eliminate the influence of reverse factors. This confirmed that the mediation analysis was unaffected by reverse causation.

### Identifying mediator metabolite phenotypes

We performed a cyclical analysis between HLA-DR-expressing CD14 + monocytes and 1,400 metabolite phenotypes utilizing the IVW method and identified 51 metabolite statistically significant metabolites (*p*-value < 0.05), as shown in Supplementary Table 6.

We identified three metabolite phenotypes with significant associations (*p*- value < 0.05) that may function as mediators affecting the outcome among the 51 metabolite phenotypes associated with IBD. These included *“Chiro-inositol levels,” “1-arachidonoyl-gpc (20:4n6) levels,”* and *“Pregnenetriol sulfate levels.”*

From preliminary mediation analysis, the mediation effects of *“1-arachidonoyl-gpc (20:4n6) levels”* and *“Pregnenetriol sulfate levels”* for HLA-DR-expressing CD14 + monocytes on IBD were contrary to the direct effects. The mediation of these two metabolites counteracted each other in the context of HLA-DR-expressing CD14 + monocytes affecting IBD. We focused solely on *“Chiro-inositol metabolites”* for subsequent mediation analysis and excluded the others. Supplementary Figs. 1 and 2 are forest plots indicating the mediation effects of *“1-arachidonoyl-gpc (20:4n6) levels”* and *“Pregnenetriol sulfate levels.”*

### Association of HLA-DR-expressing CD14 + monocytes with chiro-inositol metabolites

Genetically predicted HLA-DR-expressing CD14 + monocytes and Chiro-inositol metabolites were positively correlated based on the IVW, MR Egger, weighted median, and weighted mode methods. In the IVW method, the odds ratio (OR) was 1.05 (CI = 1.01–1.10); in MR-Egger analysis, it was 1.06 (CI = 0.98–1.15); in the weighted median method, the OR was 1.04 (CI = 0.98–1.11), and in the weighted mode methods, the OR was 1.04 (CI = 0.98–1.11). Details are shown in Figs. 3 and 4.

### Association of chiro-inositol metabolites with IBD

Similarly, employing the four methods, including IVW, a negative correlation was found between Chiro-inositol metabolites and the risk of IBD (Figs. 3 and 4). Specifically, in the IVW method, the OR was 0.92 (CI = 0.84–0.99); in the MR-Egger analysis, the OR was 0.81 (CI = 0.63–1.04); in the weighted median, the OR was 0.92 (CI = 0.83–1.02), and in the weighted mode, the OR was 0.92 (CI = 0.77–1.09). Similarly, we did not detect any interference from confounding factors using the PhenoScanner V2 tool.

### Levels of association between HLA-DR-expressing CD14 + monocytes and IBD mediated by chiro-inositol metabolites

**Fig. 5:**
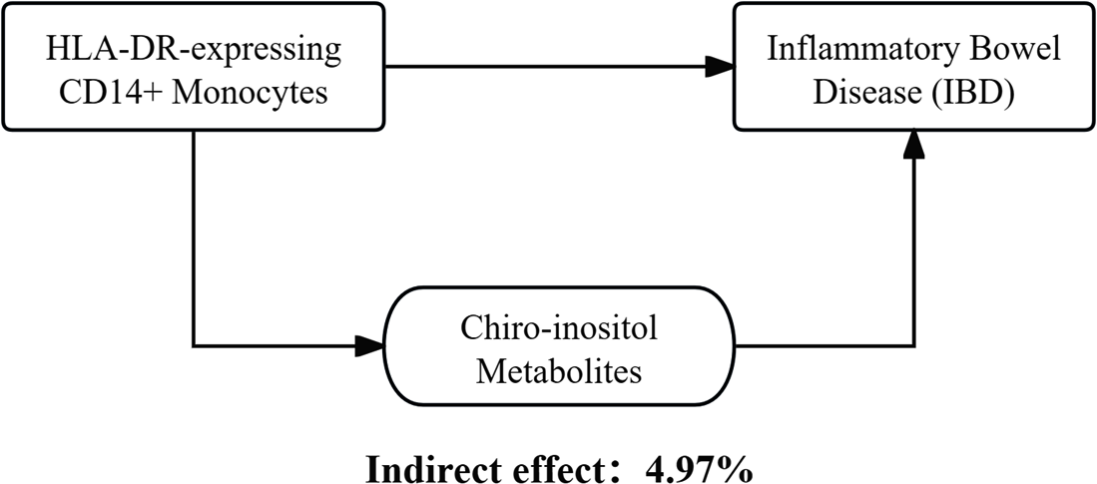
Schematic of the mediation effect of Chiro-inositol metabolites

We analyzed the mediating role of Chiro-inositol metabolites on the effect of HLA-DR-expressing CD14 + monocytes on IBD. An elevation in HLA-DR expression correspondingly increased Chiro-inositol metabolites, which, in turn, reduced the risk of IBD development. As shown in Fig. 5, mediation effect calculation suggested that Chiro-inositol metabolites reduced the risk of IBD development posed by HLA-DR-expressing CD14 + monocytes, accounting for 4.97% of the effect (level mediated: 4.97%; 95%CI= -3.2–13.2%).

### Sensitivity analysis

**Fig. 6:**
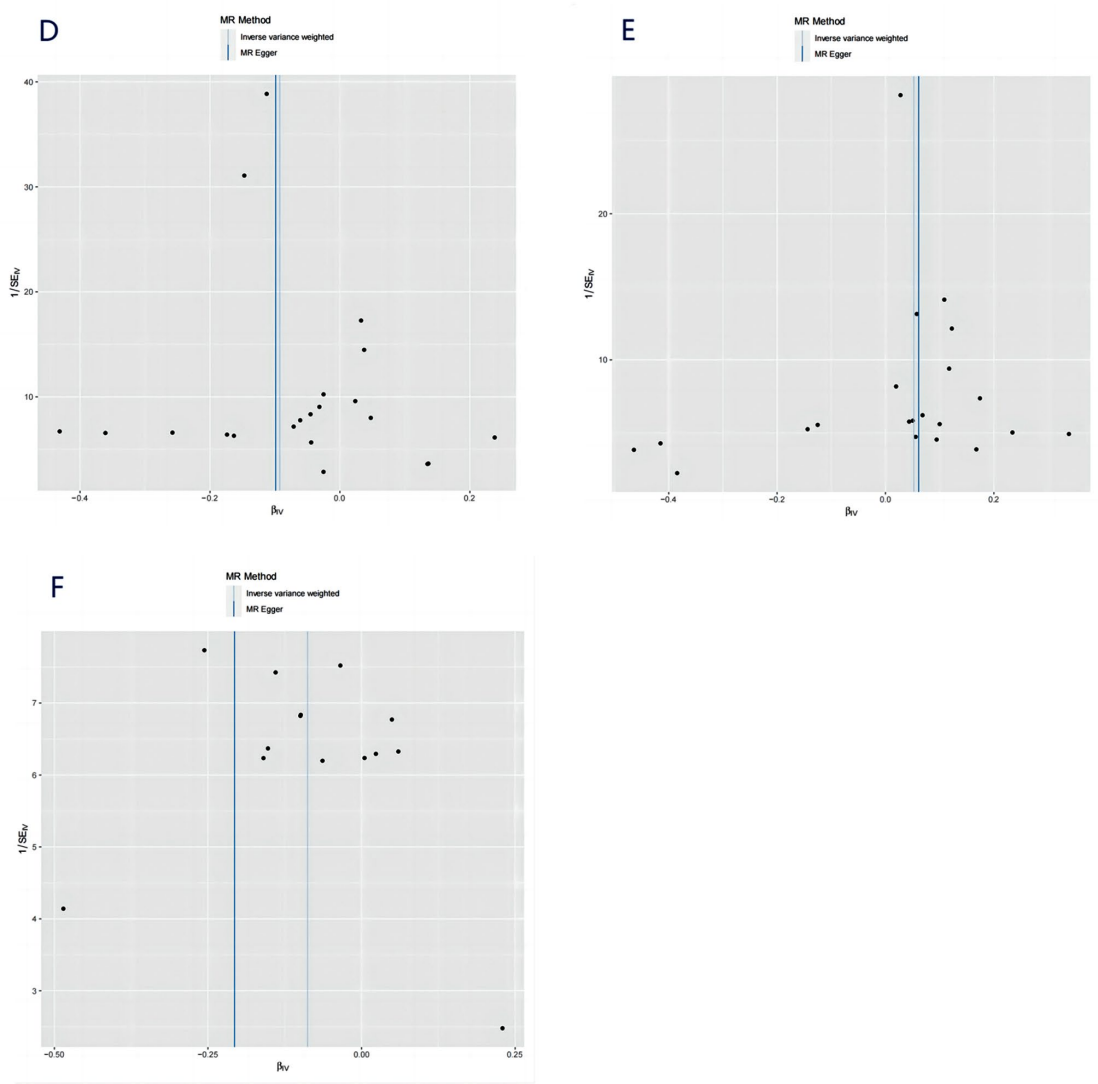
Funnel plot to assess heterogeneity. (**D**) HLA-DR-expressing CD14 + monocytes and IBD; (**E**) HLA-DR-expressing CD14 + monocytes and Chiro-inositol metabolites; (**F**) Chiro-inositol metabolites and IBD

We employed multiple sensitivity analysis methods to assess the reliability of the results. We did not detect potential horizontal pleiotropy (Supplementary Table 7) using MR-Egger regression and MR-PRESSO methods. The leave-one-out analysis indicated that no single SNP excessively affected the overall causal estimate, confirming the stability of the results (Supplementary Fig. 3). Cochran’s Q-test and funnel plot were used to detect the presence of heterogeneity (Fig. 6 and Supplementary Table 7). In our study, the MR-Egger method yielded a Q P-value < 0.05, suggesting weak heterogeneity. However, this does not undermine the overall conclusion that HLA-DR-expressing CD14 + monocytes reduced the risk of developing IBD.

## Discussion

Our study, leveraging a vast array of publicly available genetic data, pioneers the application of MR analysis to assess the causal relationships between 731 immunophenotypes, 1,400 metabolite phenotypes, and IBD. To date, no comprehensive causal analysis has covered such an extensive range of immune phenotypes, metabolite phenotypes, and IBD. We identified the causal associations between 48 immunophenotypes and IBD, with “HLA-DR on CD14 + monocytes” significantly protective effects against IBD. From 1,400 metabolite phenotypes, we recognized the role of Chiro-inositol metabolites in mediating the protective effect of HLA-DR-expressing CD14 + monocytes against IBD at 4.97%. HLA-DR-expressing CD14 + monocytes reduced the risk of IBD onset, wherein Chiro-inositol metabolites functioned indirectly.

Monocytes typically participate in immune responses and regulate inflammation [44]. HLA-DR molecules, belonging to the major histocompatibility complex (MHC) class II molecules, are pivotal in antigen presentation and immune regulation [45]. Asmussen et al. indicated that low HLA-DR expression in chronic inflammatory monocytes underscores the anti-inflammatory role of HLA-DR molecules [46]. We utilized the MR method to provide new genetic evidence for the causal relationship between HLA-DR-expressing CD14 + monocytes and IBD, confirming the role of HLA-DR-expressing CD14 + monocytes in IBD.

The mediating role of Chiro-inositol metabolites in the effect of HLA-DR-expressing CD14 + monocytes on IBD was determined. Chiro-inositol is crucial in intracellular signaling and glycolipid metabolism [47]. Supplementation with a mixture of β-glucans, inositol, and digestive enzymes enhances the quality of life for patients with IBD [48]. Our findings corroborate that Chiro-inositol can reduce the risk of IBD onset and mediate its effects through HLA-DR-expressing CD14 + monocytes.

We identified specific immune cells and metabolites associated with the risk of IBD through MR analysis, revealing numerous novel potential pathways from various immune cells to IBD. HLA-DR-expressing CD14 + monocytes exert a protective effect against IBD. This finding resonates with previous results on the role of immune cells in IBD [49], providing new evidence for the involvement of this cell type in the immune regulation of IBD. Chiro-inositol, as a mediating factor, offers a new perspective on the immune regulatory mechanisms of IBD. The regulation of myo-inositol is more feasible compared to immune cells [50]. Although the specific mechanisms of the Chiro-inositol metabolic pathway in IBD necessitate further investigation, our findings lay the theoretical groundwork for developing new therapeutic targets.

Inositol polyphosphate multikinase (IPMK) crucially regulates the homeostasis of tuft cells and indirectly affects the onset and progression of colitis [51]. Chiro-inositol, as an isomer of inositol, may exert its effects by modulating the metabolic pathways of inositol polyphosphate. We hypothesized that Chiro-inositol or its metabolites participated in modulating intestinal inflammatory responses by affecting the function of tuft cells, thus reducing the risk of IBD. Tuft cells produce endogenous intestinal opioids and prostaglandins, which are key molecules in regulating intestinal inflammation and maintaining barrier function. Therefore, Chiro-inositol may play a protective role by regulating the production of these molecules.

However, some limitations of our study warrant further consideration. First, although MR analysis can effectively reduce the effect of confounding factors, all potential confounders, especially unknown genetic variants that might influence the study outcomes, cannot be excluded. Second, although our study is based on data from large-scale GWASs, the samples were primarily from European populations, so the results cannot be generalized to populations of other ethnicities or geographic regions. The mediating role of Chiro-inositol metabolites between HLA-DR-expressing CD14 + monocytes and IBD is relatively small, suggesting the presence of other undiscovered mediating mechanisms.

Based on our findings, subsequent research should explore the mechanisms of HLA-DR-expressing CD14 + monocyte action in immune regulation, and preventing and treating IBD by modulating Chiro-inositol metabolites. Investigating other biological pathways related to Chiro-inositol metabolism can reveal new therapeutic targets. Ultimately, validating these findings across diverse populations and environmental factors can support the development of personalized treatment strategies.

## Conclusion

In summary, we identified the protective effect of HLA-DR-expressing CD14 + monocytes against IBD, with a minor role of Chiro-inositol metabolites. However, most of the effects of HLA-DR-expressing CD14 + monocytes on IBD remain unclear. Further research should investigate other potential risk factors that may serve as mediators. More attention must be paid on the effect of HLA-DR-expressing CD14 + monocytes on patients with IBD.

### Electronic supplementary material

Below is the link to the electronic supplementary material.

Supplementary Material 1

Supplementary Material 2

Supplementary Material 3

## Data Availability

The present study involves summary-level data that have been made publically available. Summary data from genome-wide association studies on inflammatory bowel disease are available at https://risteys.finregistry.fi/endpoints/K11_IBD_STRICT. We collected dataon immune cells and metabolites. Data on 731 immunophenotypes are available from https://gwas.mrcieu.ac.uk/. Detailed GWAS IDs are provided in Supplementary Table 1. Data on 1,400 metabolite phenotypes were obtained from https://www.ebi.ac.uk/gwas/. Detailed IDs are provided in Supplementary Table 2.
