# Supplementary material for: Mediating role of chiro-inositol metabolites on the effects of HLA-DR-expressing CD14 + monocytes in inflammatory bowel disease
Source: BMC Gastroenterol. 2024 Jun 17;24:200. doi: 10.1186/s12876-024-03271-2 (PMC11181584; doi:10.1186/s12876-024-03271-2)
Supplement: Supplementary file 3 — Supplementary Material 3 [file 12876_2024_3271_MOESM3_ESM.pdf]

Exposure	Outcome	Method	No.SNP	OR(95%CI)	P
HLA-DR-expressing CD14+ Monocytes on IBD					
	IBD	MR Egger	21	0.906 (0.838 to 0.979)	2.19e-02
	IBD	Weighted median	21	0.894 (0.853 to 0.938)	5.05e-06
	IBD	Inverse variance weighted	21	0.912 (0.876 to 0.948)	3.86e-06
	IBD	Weighted mode	21	0.887 (0.851 to 0.925)	1.52e-05
HLA-DR-expressing CD14+ Monocytes on 1-arachidonoyl-gpc (20:4n6) levels					
	1-arachidonoyl-gpc (20:4n6) levels	MR Egger	21	0.975 (0.920 to 1.034)	4.14e-01
	1-arachidonoyl-gpc (20:4n6) levels	Weighted median	21	0.963 (0.919 to 1.008)	1.06e-01
	1-arachidonoyl-gpc (20:4n6) levels	Inverse variance weighted	21	0.965 (0.934 to 0.998)	3.98e-02
	1-arachidonoyl-gpc (20:4n6) levels	Weighted mode	21	0.960 (0.916 to 1.005)	9.82e-02
1-arachidonoyl-gpc (20:4n6) levels on IBD					
	IBD	MR Egger	27	0.876 (0.810 to 0.947)	2.58e-03
	IBD	Weighted median	27	0.871 (0.824 to 0.921)	1.26e-06
	IBD	Inverse variance weighted	27	0.898 (0.854 to 0.944)	2.72e-05
	IBD	Weighted mode	27	0.875 (0.829 to 0.924)	5.84e-05

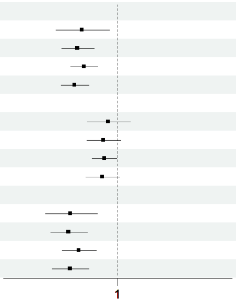

Supplementary Figure 1. Forest plot shows the causal relationships among HLA-DR-expressing CD14+ monocytes, 1-arachidonoyl-gpc (20:4n6) levels, and IBD.

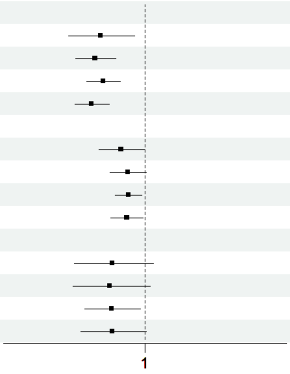

Exposure	Outcome	Method	No.SNP	OR(95%CI)	P
HLA-DR-expressing CD14+ Monocytes on IBD					
	IBD	MR Egger	21	0.906 (0.838 to 0.979)	2.19e-02
	IBD	Weighted median	21	0.894 (0.853 to 0.938)	5.05e-06
	IBD	Inverse variance weighted	21	0.912 (0.876 to 0.948)	3.86e-06
	IBD	Weighted mode	21	0.887 (0.851 to 0.925)	1.52e-05
CD14+ Monocytes on Pregnenetriol sulfate					
	Pregnenetriol sulfate	MR Egger	21	0.949 (0.902 to 0.999)	6.18e-02
	Pregnenetriol sulfate	Weighted median	21	0.963 (0.926 to 1.003)	6.70e-02
	Pregnenetriol sulfate	Inverse variance weighted	21	0.965 (0.938 to 0.994)	1.71e-02
	Pregnenetriol sulfate	Weighted mode	21	0.962 (0.928 to 0.997)	4.60e-02
Pregnenetriol sulfate on IBD					
	IBD	MR Egger	35	0.930 (0.850 to 1.018)	1.27e-01
	IBD	Weighted median	35	0.926 (0.848 to 1.011)	8.54e-02
	IBD	Inverse variance weighted	35	0.930 (0.872 to 0.990)	2.41e-02
	IBD	Weighted mode	35	0.931 (0.864 to 1.003)	6.83e-02

Supplementary Figure 2. Forest plot shows the causal relationships among HLA-DR-expressing CD14+ monocytes, Pregnenetriol sulfate metabolites, and IBD.

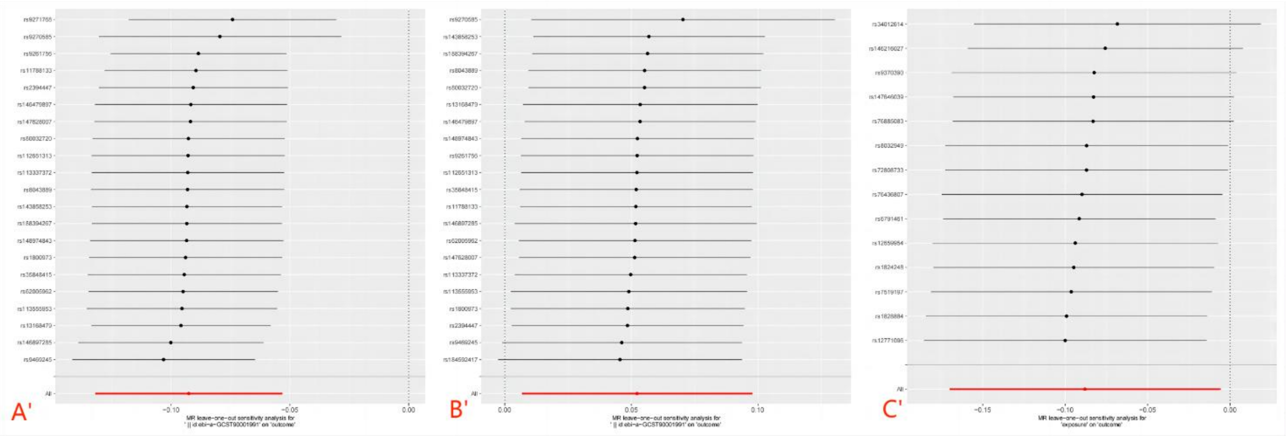

Supplementary Figure 3. Leave-one-out plot to visualize causal effect when leaving one SNP out. (A') HLA-DR-expressing CD14+ monocytes and IBD; (B') HLA-DR-expressing CD14+ monocytes and Chiro-inositol metabolites; (C') Chiro-inositol metabolites and IBD.

Supplementary Table 3. Immunophenotypes with significant associations on IBD.

id	pvalue
ebi-a-GCST90001395	0.029643294
ebi-a-GCST90001438	0.049625582
ebi-a-GCST90001447	0.023043873
ebi-a-GCST90001449	0.046569161
ebi-a-GCST90001459	0.019589818
ebi-a-GCST90001461	0.044410038
ebi-a-GCST90001463	0.045136641
ebi-a-GCST90001476	0.016000314
ebi-a-GCST90001490	0.027469832
ebi-a-GCST90001513	0.010419574
ebi-a-GCST90001516	0.031893161
ebi-a-GCST90001526	0.019403257
ebi-a-GCST90001530	0.0193686
ebi-a-GCST90001541	0.015580321
ebi-a-GCST90001594	0.006415244
ebi-a-GCST90001648	0.005516676
ebi-a-GCST90001649	0.01599666
ebi-a-GCST90001650	0.025303115
ebi-a-GCST90001658	3.03E-05
ebi-a-GCST90001682	0.039790448
ebi-a-GCST90001695	0.025075627
ebi-a-GCST90001723	0.029844723
ebi-a-GCST90001725	0.036251638
ebi-a-GCST90001727	0.036821663
ebi-a-GCST90001730	0.04381148
ebi-a-GCST90001740	0.001295238
ebi-a-GCST90001741	0.022689908
ebi-a-GCST90001781	0.04414253
ebi-a-GCST90001797	0.048496158

id	pvalue
ebi-a-GCST90001831	0.005668547
ebi-a-GCST90001891	0.038691055
ebi-a-GCST90001922	0.023936708
ebi-a-GCST90001968	0.019387206
ebi-a-GCST90001984	0.04372632
ebi-a-GCST90001988	0.000205729
ebi-a-GCST90001991	3.86E-06
ebi-a-GCST90001995	0.03502795
ebi-a-GCST90001997	0.020854808
ebi-a-GCST90002010	0.023044349
ebi-a-GCST90002073	0.005678076
ebi-a-GCST90002074	0.001649806
ebi-a-GCST90002097	0.043452384
ebi-a-GCST90002105	0.000114561
ebi-a-GCST90002106	0.000933176
ebi-a-GCST90002109	0.030056062
ebi-a-GCST90002111	0.039600005
ebi-a-GCST90002112	0.012780292
ebi-a-GCST90002117	0.030791219

Abbreviations: IBD, Inflammatory Bowel Disease.

Supplementary Table 4. The results of the MR Steiger test.

**Exposure**	**Outcome**	**R^2 for exposure**	**R^2 for outcome**	**Correct causal direction**	**$P_{steiger}$**
HLA-DR on CD14+ monocyte	IBD	1.34×10^{-1}	1.75×10^{-2}	TRUE	3.72×10^{-37}

Abbreviations: IBD, Inflammatory Bowel Disease.

Supplementary Table 5. Results of MR Analysis of IBD on HLA-DR-expressing CD14+ monocytes.

method	nsnp	b	se	pval	lo_ci	up_ci	or	or_lci95	or_uci95
MR Egger	44	-0.19114	0.13853	0.174966	-0.46265	0.080382	0.826021	0.629611	1.083701
Weighted median	44	0.000941	0.050241	0.985053	-0.09753	0.099414	1.000942	0.907074	1.104523
Inverse variance weighted	44	-0.01472	0.055163	0.789612	-0.12284	0.093401	0.98539	0.884407	1.097902
Simple mode	44	0.005389	0.085776	0.950193	-0.16273	0.173511	1.005404	0.849819	1.189474
Weighted mode	44	0.01264	0.066332	0.849773	-0.11737	0.142651	1.01272	0.889255	1.153327

Abbreviations: IBD, Inflammatory Bowel Disease.

Supplementary Table 6. Results of IVW Analysis of HLA-DR-expressing CD14+ monocytes on 1400 metabolite phenotypes (*p*-value < 0.05).

imc	dxw	pvalue
ebi-a-GCST90001991	GCST90199840	0.04009519
ebi-a-GCST90001991	GCST90199843	0.024163659
ebi-a-GCST90001991	GCST90200327	0.01446815
ebi-a-GCST90001991	GCST90200332	0.020895738
ebi-a-GCST90001991	GCST90200355	0.047006196
ebi-a-GCST90001991	GCST90200617	0.032184804
ebi-a-GCST90001991	GCST90200597	0.027078206
ebi-a-GCST90001991	GCST90200964	0.029581164
ebi-a-GCST90001991	GCST90200966	0.01453444
ebi-a-GCST90001991	GCST90200971	0.029176955
ebi-a-GCST90001991	GCST90200135	0.040963663
ebi-a-GCST90001991	GCST90200435	0.035153693
ebi-a-GCST90001991	GCST90199860	0.02617388
ebi-a-GCST90001991	GCST90200067	0.041206795
ebi-a-GCST90001991	GCST90200026	0.023947373
ebi-a-GCST90001991	GCST90199983	0.046293352
ebi-a-GCST90001991	GCST90199660	0.000334096
ebi-a-GCST90001991	GCST90199725	0.003316337
ebi-a-GCST90001991	GCST90199673	0.037305733
ebi-a-GCST90001991	GCST90199635	0.008308167
ebi-a-GCST90001991	GCST90199918	0.0430696
ebi-a-GCST90001991	GCST90199917	0.043550863
ebi-a-GCST90001991	GCST90199931	0.030920558
ebi-a-GCST90001991	GCST90199830	0.039299097
ebi-a-GCST90001991	GCST90200584	0.035311366
ebi-a-GCST90001991	GCST90200693	0.025061044
ebi-a-GCST90001991	GCST90200769	0.048359742

imc	dxw	pvalue
ebi-a-GCST90001991	GCST90200721	0.027825605
ebi-a-GCST90001991	GCST90200732	0.044609633
ebi-a-GCST90001991	GCST90200733	0.007819628
ebi-a-GCST90001991	GCST90199788	0.039848074
ebi-a-GCST90001991	GCST90199764	0.040237074
ebi-a-GCST90001991	GCST90199775	0.043480271
ebi-a-GCST90001991	GCST90200524	0.030202397
ebi-a-GCST90001991	GCST90200682	0.012745761
ebi-a-GCST90001991	GCST90200315	0.042034682
ebi-a-GCST90001991	GCST90200484	0.048427025
ebi-a-GCST90001991	GCST90200947	0.004873113
ebi-a-GCST90001991	GCST90200949	0.021511291
ebi-a-GCST90001991	GCST90200962	0.020459129
ebi-a-GCST90001991	GCST90200844	0.033533584
ebi-a-GCST90001991	GCST90200940	0.029510051
ebi-a-GCST90001991	GCST90200868	0.035532371
ebi-a-GCST90001991	GCST90200238	0.025962399
ebi-a-GCST90001991	GCST90200241	0.017080932
ebi-a-GCST90001991	GCST90200838	0.026281803
ebi-a-GCST90001991	GCST90200819	0.043590304
ebi-a-GCST90001991	GCST90200821	0.035936304
ebi-a-GCST90001991	GCST90200823	0.030422738
ebi-a-GCST90001991	GCST90200824	0.011436817
ebi-a-GCST90001991	GCST90200832	0.045052363

Supplementary Table 7. Heterogeneity and pleiotropy in MR analyses.

EXPOSURE	OUTCOME	METHOD	NO. OF SNPs	HETEROGENEITY			HORIZONTAL PLEIOTROPY			
							MR-EGGER REGRESSION			MR-PRESSO
				Q	Q DEGREES OF FREEDOM	Q P-VALUE	EGGER INTERCEPT	SE	P-VALUE	GLOBAL TEST P-VALUE
HLA-DR on CD14+ monocyte	IBD	MR Egger	21	30.68	19	0.04	0.003	0.014	0.855	0.059
HLA-DR on CD14+ monocyte	IBD	Inverse variance weighted	21	30.74	20	0.06				
HLA-DR on CD14+ monocyte	Chiro-inositol levels	MR Egger	21	17.13	19	0.58	-0.004	0.014	0.788	0.692
HLA-DR on CD14+ monocyte	Chiro-inositol levels	Inverse variance weighted	21	17.21	20	0.64				
Chiro-inositol levels	IBD	MR Egger	14	7.32	12	0.84	0.018	0.018	0.344	0.834
Chiro-inositol levels	IBD	Inverse variance weighted	14	8.29	13	0.82				

Abbreviations: IBD, Inflammatory Bowel Disease.
